# Bottom-up proteomics suggests an association between differential expression of mitochondrial proteins and chronic fatigue syndrome

**DOI:** 10.1038/tp.2016.184

**Published:** 2016-09-27

**Authors:** F Ciregia, L Kollipara, L Giusti, R P Zahedi, C Giacomelli, M R Mazzoni, G Giannaccini, P Scarpellini, A Urbani, A Sickmann, A Lucacchini, L Bazzichi

**Affiliations:** 1 Department of Pharmacy, University of Pisa, Pisa, Italy; 2 Leibniz-Institut für Analytische Wissenschaften—ISAS, Dortmund, Germany; 3 Division of Rheumatology, Department of Clinical and Experimental Medicine, University of Pisa, Pisa, Italy; 4 Division of Psychiatry, Department of Clinical and Experimental Medicine, University of Pisa, Pisa, Italy; 5 Istituto di Biochimica e Biochimica Clinica, Università Cattolica, Rome, Italy; 6 Proteomics and Metabonomics Unit, IRCCS-Fondazione Santa Lucia, Rome, Italy; 7 Department of Chemistry, College of Physical Sciences, University of Aberdeen. Aberdeen, UK; 8 Medizinische Fakultät, Medizinische Proteom-Center, Ruhr-Universität Bochum, Bochum, Germany

## Abstract

Chronic fatigue syndrome (CFS) is a debilitating and complex disorder characterized by unexplained fatigue not improved by rest. An area of investigation is the likely connection of CFS with defective mitochondrial function. In a previous work, we investigated the proteomic salivary profile in a couple of monozygotic twins discordant for CFS. Following this work, we analyzed mitochondrial proteins in the same couple of twins. Nano-liquid chromatography electrospray ionization mass spectrometry (nano-LC-MS) was used to study the mitochondria extracted from platelets of the twins. Subsequently, we selected three proteins that were validated using western blot analysis in a big cohort of subjects (*n*=45 CFS; *n*=45 healthy), using whole saliva (WS). The selected proteins were as follows: aconitate hydratase (ACON), ATP synthase subunit beta (ATPB) and malate dehydrogenase (MDHM). Results for ATPB and ACON confirmed their upregulation in CFS. However, the MDHM alteration was not confirmed. Thereafter, seeing the great variability of clinical features of CFS patients, we decided to analyze the expression of our proteins after splitting patients according to clinical parameters. For each marker, the values were actually higher in the group of patients who had clinical features similar to the ill twin. In conclusion, these results suggest that our potential markers could be one of the criteria to be taken into account for helping in diagnosis. Furthermore, the identification of biomarkers present in particular subgroups of CFS patients may help in shedding light upon the complex entity of CFS. Moreover, it could help in developing tailored treatments.

## Introduction

Chronic fatigue syndrome (CFS) is a debilitating and complex disorder characterized by unexplained fatigue not improved by rest, that lasts longer than 6 months, and that may be aggravated by physical or mental activity. In CFS, fatigue is also characterized by rheumatological, and neuropsychiatric symptoms such as joint pain, aching or stiff muscles, concentration and memory problems, sleep disruption, sore throat, headaches and so on.^1, 2^ In 2011, the International Consensus Criteria have been defined to identify CFS by revising the Canadian definition released in 2003;^3^ however, at present, no laboratory tests can be used to objectively diagnose CFS. Therefore, the diagnosis of CFS remains a clinical endeavor and can be made after the exclusion of medical conditions that may explain the prolonged fatigue as well as a number of psychiatric diagnoses (for example, bipolar disorder, eating disorders, psychotic disorders and melancholic depression).

This difficulty in defining CFS, due to the lack of diagnostic markers, complicates epidemiological studies. Prevalence varies from as low as 0.2% to as high as 6.41%,^4, 5^ with an incidence rate that differed strongly with age, with a first peak in the age group 10–19 years and a second peak in the age group 30–39 years.^6, 7^

Up till now, the etiology of CFS remains unclear; it is likely that CFS is a heterogeneous disorder that could be explained by a multifactorial model where genetic, infectious, neuroendocrine and psychological factors converge.

One of the most common proposed causative agent is a viral infection, and vaccines have been also indicated to contribute to the development of CFS; however, data are controversial.^8, 9, 10, 11, 12^ In addition, several immune abnormalities have been reported in CFS patients; however, many of the observed abnormalities were not confirmed by others, resulting in inconsistent findings across studies.^13, 14^ Hence, there is a clear need to enhance CFS understanding.

Another area of investigation is the likely connection of CFS with defective mitochondrial function. Recently, studies on metabolism and CFS suggest irregularities in energy metabolism and oxidative stress metabolism.^15^ In particular, it is increasingly recognized that CFS can be underpinned by mitochondrial dysfunctions;^16, 17, 18^ moreover, fatigue is a prominent symptom in patients with mitochondrial disease.^19^ This is an attractive hypothesis, as a metabolic alteration at the mitochondrial level could entail an energy deficiency and explain the peculiar fatigue.

Hitherto, it has often been suggested that CFS may have a genetic predisposition; therefore, twin studies supply the best matched controls in a case–control research. Thereby, in a previous work, we investigated the proteomic salivary profile in a couple of monozygotic twins discordant for CFS.^20^ In particular, our results highlighted the involvement of inflammatory response in CFS pathogenesis. Following the encouraging results obtained with this preliminary study, we sought to improve our knowledge about mechanisms of CFS by analyzing mitochondrial proteins in the same couple of monozygotic twins discordant for CFS. The expectation was that our findings could be able to provide evidence of a mitochondrial role in CFS pathogenesis.

With this purpose, we isolated mitochondria from platelets. Platelets are circulating blood cells that are essential not only in hemostasis and coagulation but also in innate and adaptive immunity.^21^ Platelets have functionally active mitochondria, whose involvement in cell physiology and in pathological conditions has been increasingly demonstrated.^21, 22^ The recent assessments of mitochondrial function in platelets offer several advantages for diagnostic purposes, such as their abundance in peripheral blood, the relative ease of obtaining platelets, their limited complexity,^23^ the considerable number of mitochondria contained in platelets and the ability to make repeated measurements for each patient.^21^

Platelets have already been used in investigations of mitochondrial involvement in some disorders, for example, in sepsis,^24^ neurodegenerative diseases^25, 26, 27^ or diabetes.^28, 29^ However, these studies were limited to mitochondria DNA analysis and electron transfer activities, whereas no investigation has been performed on the total mitochondrial protein content. Therefore, we analyzed with nano-liquid chromatography electrospray ionization mass spectrometry (nano-LC-MS) the mitochondria extracted from platelets of the same couple of monozygotic twins discordant for CFS who have been the subjects of our previous work. Subsequently, the most promising biomarkers were validated by western blot analysis in a big cohort of patients, using whole saliva (WS), whose sampling is a noninvasive, simple, safe and stress-free procedure that can be easily applied to large groups of subjects.

Seeing the vast heterogeneity of the disease, the aim was to suggest biomarkers to stratify CFS cases, rather than selecting some biomarkers for identifying CFS. This can help to customize intervention.

## Materials and methods

### Study design

This translational case–control study was subdivided into three different steps. The first ‘discovery' phase was aimed at characterizing the mitochondrial proteomic profile of a patient suffering from CFS in comparison with his healthy monozygotic twin. This purpose was achieved by using a proteomics approach: nano-LC-MS. The second ‘exploratory' phase was aimed at investigating the changing proteins; we applied the Ingenuity Pathway Analysis (IPA) Knowledge base. This platform enabled to visualize the potential interactions between the identified biomarker signatures in mitochondria helping us to select some candidates for the ‘validation' phase. Selected proteins were first searched in WS of the twin brothers and, once the differences have been confirmed, the validation was widened to a larger number of patients and controls. Finally, we have amplified our analysis looking closely at clinical data, with the specific aim to investigate how the biomarkers correlate with patients' clinical parameters. This leads us to identify mitochondrial proteins useful in subtyping patients with CFS.

### Patients

The recruitment of the subjects was managed by the Division of Rheumatology, Department of Clinical and Experimental Medicine, University of Pisa, Pisa, Italy. This study has been approved by the local Ethics Committee, and an informed consensus was obtained for diagnostic or clinical purposes.

The twins of the previous study^20^ were enrolled to collect peripheral venous blood samples for the purpose of ‘discovery' phase, and saliva for the validation. Samples, both blood and WS, were collected three times from the twins. In addition, a total of 45 patients affected by CFS were consecutively recruited for the ‘validation' phase; mean age 38.6±11.9 years (mean±s.d.; M±s.d.; Table 1). Forty-five healthy subjects, with similar mean age (36.4±10.9, M±s.d.; 25 females, 20 males) and demographic characteristics, were included as controls (see Supplementary Methods 1). The patients were classified based on the classification criteria of Fukuda *et al.*^30^

The patients performed a rheumatologic visit with routine clinical evaluation of medical history. The subjects completed the Fibromyalgia Impact Questionnaire (FIQ), in which pain and fatigue severity were assessed with 10-cm Visual Analog Score Scale (VAS) and short form-36. Fatigue was assessed also by means of the Functional Assessment of Chronic Illness Therapy-Fatigue Scale (FACIT). We considered the VAS from the FIQ asking whether the patient felt rested upon awaking during the last week. In order to assess the presence of psychiatric concomitant disorders, patients had a psychiatric evaluation based on the administration of the Structured Clinical Interview for Diagnostic and Statistical Manual of Mental Disorders, 4th Edition, axis-I disorders.^31^

To better classify the subjects the following hematochemical parameters were evaluated:

- Immunological parameters: antinuclear antibodies, extractable nuclear antigen antibodies, rheumathoid factor (Ra test) and anticardiolipin antibodies.
- Hormone profile: cortisole and adrenocorticotropic hormone, serotonin, growth hormone, insulin-like growth factor-1 and vitamin D3.
- Cytokine profile: tumor necrosis factor (TNF)-α, interferon (IFN)-γ, interleukin (IL)-6, IL-10, IL-2, IL-8 and IL-1b.

Table 1 summarizes clinical data.

### WS collection

Salivary samples were collected from patients (*n*=45) and controls (*n*=45) with a saliva collector sponge (Surescreen Diagnostics, Derby, UK). WS samples were collected early in the morning (between 0800 and 1100 hours) according to a standard protocol.^32^ No evidence of oral pathologies or inflammatory processes was observed. The collected saliva was immediately centrifuged at 17 000 *g* for 20 min at 4 °C to yield clear samples. The resulting supernatants were stored at −80 °C. Protein amounts of samples were determined using the Bio-Rad DC-protein assay (Bio-Rad, Hercules, CA, USA). Bovine serum albumin was used as a standard.

### Platelet collection and mitochondrial enrichment

Blood samples were drawn from an antecubital vein using a 19-gauge needle and the first 2.5 ml were discarded. All samples were collected in Vacutainer tubes (Becton Dickinson, San Jose, CA, USA), containing 3.8% sodium citrate as anticoagulant.

Peripheral venous blood sample (25 ml) was drawn from the subjects and placed in proper collecting tubes with heparin. The samples were immediately processed to obtain platelets. Blood was centrifuged for 15 min at 300 *g* to obtain a platelet-rich plasma and a pellet-containing red cells, granulocytes and lymphocytes. Platelets were precipitated from platelet-rich plasma by centrifugation at 3000 *g* for 10 min and resuspended in 0.9% NaCl; an aliquot of 300 μl was stored for normalization, whereas the remaining was used for mitochondrial enrichment.

Isolation of mitochondrial fractions from human platelets was performed by differential centrifugation. The platelets were centrifuged for 10 min at 3000 *g*. A volume of cold lysis buffer (10 mM Tris-HCl pH 7.6), depending on the size of the pellet, was added and kept 7 min on ice stirring occasionally. After centrifugation for 10 min at 1000 *g* we obtained unbroken cells (P1). The supernatant, containing mitochondria, was immediately centrifuged for 20 min at 9000 *g* obtaining mitochondrial fraction M1. P1 was further resuspended in cold lysis buffer, and kept for 7 min on ice stirring occasionally. After a centrifugation for 10 min at 1000 *g*, the resulting supernatant was immediately centrifuged for 20 min at 9000 *g* obtaining mitochondrial fraction M2. M1 and M2 were immediately resuspended in cold buffer (10 mM Tris-HCl pH 7.4, 2 mM EDTA, 250 mM Sucrose) and combined. Protein amounts were determined using the Bio-Rad DC-protein assay (Bio-Rad). Bovine serum albumin was used as a standard.

The integrity of mitochondria was assayed by measuring the activity of cytochrome oxidase. Western blot (WB) analysis was performed to evaluate the purity of the mitochondria as described in Supplementary Method 2. Blood was collected three times, and mitochondria obtained from each experiments were pooled and analyzed in triplicate by LC-MS-based label-free quantification.

### LC-MS-based label-free quantification

#### Sample preparation for LC-MS

Platelets, kept for normalization, and mitochondria were analyzed by label-free LC-MS analysis. Samples were resuspended in the lysis buffer (50 mM Tris pH 7.8, 150 mM NaCl, 1% sodium dodecylsulphate (SDS)) supplemented with protease inhibitors and phosphatase inhibitors (Roche, Mannheim, Germany; 1 tablet per 10 ml buffer) and sonicated. After centrifugation (18 000 *g* for 15 min at 4 °C), the protein amount of the lysate was determined using the bicinchoninic acid protein assay according to the manufacturer's instructions (Pierce BCA Protein Assay Kit, Thermo Scientific, Bremen, Germany). Cysteines were reduced with 10 mM dithiothreitol at 56 °C for 30 min and the free thiols were carbamidomethylated with 30 mM iodoacetamide at room temperature for 30 min in the dark.

Sample cleaning and proteolysis were based on the filter-aided sample preparation protocol.^33, 34^ Briefly, cell lysates corresponding to 135 μg of protein were diluted up to 500 μl with freshly prepared 8 M urea/100 mM Tris-HCl (pH 8.5) buffer.^35^ Diluted samples were placed on the centrifugal devices (PALL microsep, 30 KDa cutoff) and were centrifuged at 13 800 *g* at room temperature for 20 min. All the following centrifugation steps were performed under similar conditions. To eliminate residual SDS, three washing steps were carried out using 100 μl of 8.0 M urea/100 mM Tris-HCl buffer, pH 8.5 and finally for the buffer exchange, the devices were washed three times with 100 μl of 50 mM NH_4_HCO_3_ buffer, pH 7.8. To the concentrated proteins, 100 μl of proteolysis buffer comprising trypsin (Promega, Madison, WI, USA; 1:25 *w*/*w*, enzyme to protein), 0.2 M guanidine hydrochloride and 2 mM CaCl_2_ in 50 mM NH_4_HCO_3_, pH 7.8, were added and incubated at 37 °C for 14 h. The generated tryptic peptides were recovered by centrifugation with 50 μl of 50 mM NH_4_HCO_3_, followed by 50 μl of ultrapure water. Finally, the peptides were acidified with 10% trifluoroacetic acid to pH<3, and the digests were quality-controlled as described previously.^36^

#### LC-MS analysis

Each condition was measured in triplicate (500 ng each) using an Ultimate 3000 nanoRSLC system coupled to an Orbitrap Elite mass spectrometer (both Thermo Scientific). Samples were analyzed in a randomized order to minimize systematic errors. Briefly, peptides were preconcentrated on a 100 μm × 2 cm C18 trapping column for 10 min using 0.1% trifluoroacetic acid at a flow rate of 20 μl min^−1^, followed by separation on a 75 μm × 50 cm C18 analytical column (both Pepmap, Thermo Scientific) with a 90-min LC gradient ranging from 3 to 42% of buffer B (84% acetonitrile, 0.1% formic acid) at a flow rate of 250 nl min^−1^. MS survey scans were acquired in the Orbitrap from *m*/*z* 300 to 1500 at a resolution of 60 000 using the polysiloxane ion at *m*/*z* 371.101236 as lock mass.^37^ The 15 most intense signals were subjected to collision-induced dissociation in the ion trap, taking into account a dynamic exclusion of 30 s. Collision-induced dissociation spectra were acquired with a normalized collision energy of 35% and an activation time of 10 ms. Automatic gain control target values were set to 1 × 10^6^ for Orbitrap MS and 1x10^4^ for ion trap MS/MS scans, and maximum injection times were set to 100 ms for both full MS and MS/MS scans.

### Label-free data analysis

Label-free quantification of the acquired MS data was performed using the Progenesis LC-MS software from Nonlinear Dynamics (Newcastle upon Tyne, UK) version 4.1. MS data-processing, including alignment of raw data, selection of the reference LC-MS run and peak picking, was done automatically by Progenesis. The features within retention time and *m*/*z* windows from 0 to 90 min and 300–1500 *m*/*z* with charge states +2, +3 and +4 were considered for peptide statistics, analysis of variance and principal component analysis. Spectra were exported as peak lists, searched against a concatenated target/decoy version of the human Uniprot database, (downloaded on 11 December 2013, containing 20 273 target sequences) using Mascot 2.4 (Matrix Science, Boston, MA, USA). Trypsin was selected as enzyme with a maximum of two missed cleavage sites, carbamidomethylation of Cys was set as fixed and oxidation of Met was selected as variable modification. MS and MS/MS tolerances were set to 10 p.p.m. and 0.5 Da, respectively. Peptide Shaker version 0.28.0 (ref. 38) was used for processing peptide-spectrum matches obtained from Mascot and filtering the results at a false discovery rate of 1% on the peptide-spectrum match, peptide and protein level. Results were exported using the advanced Peptide Shaker features, allowing direct re-import of the quality-controlled data into Progenesis. Only proteins that were quantified with ⩾2 unique peptides were considered for further analysis. The signal intensities of each protein present in the mitochondrial fraction were normalized on the total content of the same proteins obtained from the analysis of total platelets.

### Signaling pathway analysis

Proteins differentially expressed were functionally analyzed through the use of QIAGEN's IPA (Ingenuity System, QIAGEN, Redwood City, CA, USA) to select candidates for validation.

All differentially expressed proteins with a fold variation in CFS greater than 2.0 with respect to control were included in bioinformatic analysis to identify molecular functions most strongly associated with the protein list. Supplementary Method 3 gives more details about this analysis.

### WB for validation

To validate the expression changes in WS, we used WB analysis. The selected proteins were as follows: aconitate hydratase (ACON), ATP synthase subunit beta (ATPB) and malate dehydrogenase (MDHM). For ACON and MDHM, we loaded 30 μg of proteins and 10 μg for ATPB.

Proteins were resolved by 12% SDS-PAGE gels and transferred on nitrocellulose membranes as previously described.^39^ ACON and MDHM antibodies have been used at 1:1000 dilution (Cell Signaling Technology, Danvers, MA, USA), whereas the ATPB antibody (Santa Cruz Biotechnology, Dallas, TX, USA) has been used at 1:200 dilution. A horseradish peroxidase-conjugated goat anti-rabbit (Stressgen, Farmingdale, NY, USA) was used as a secondary antibody at 1:10 000 dilution. Immunoblots were developed using the enhanced chemiluminescence detection system. The chemiluminescent images were acquired using LAS4010 (GE Health Care, Uppsala, Sweden). The antigen-specific bands were quantified using the Image Quant-L (GE Health Care) software. In order to normalize the optical density of immune-reactive bands, the optical density of total proteins was calculated. Therefore, immediately after WB, the membranes were stained with 1 mM RuBP as previously described.^39^

### Statistical analysis

All data are presented as mean±s.e.m. Comparisons between groups were performed using the Mann–Whitney *U-*test for non-normal data. A *P*-value<0.05 was considered significant. Linear regression analysis was used to determine the correlation among levels of different biomarkers. Logistic regression was used to determine the weight given to each marker and then to calculate a specific formula to provide a combined risk index. To estimate whether this marker combination might increase the marker's performance in CFS diagnosis, receiver operating characteristic (ROC) curves were plotted, and the areas under curves (AUCs) were calculated with their 95% confidence intervals using standard techniques to evaluate sensitivity and specificity of each marker and their combination. Statistical analyses were performed with GraphPad Prism (San Diego, CA, USA) and SPSS (SPSS/PC Statistical Package for the Social Science, update for 10.1., SPSS, Chicago, IL, USA, 2000).

### Clinical correlations

To determine the statistical correlations among putative biomarkers and serological measures, the Spearman's rank correlation coefficient was calculated, a non-parametric measure of correlation based on data ranks. A *P*-value<0.05 was considered significant. Clinical correlations were performed with SPSS.

## Results

### Proteomic analysis

In order to profile the mitochondrial proteins in CFS, we purified mitochondria from platelets of the same couple of monozygotic twins, discordant for CFS, which was investigated in our previous work.^20^ To evaluate the quality of the isolated mitochondria, enzymatic assay and WB analysis were employed (see Supplementary Results 1), confirming that we reached satisfactory mitochondrial enrichment (Supplementary Figure). We obtained a mitochondrial fraction of 450 μg of proteins starting roughly from 5 × 10^9^ platelets.

Moreover, with the aim to overcome the limit of reproducibility in the mitochondrial preparation, experiments were performed three times. Mitochondria obtained from each of the three experiments were pooled together and analyzed in triplicate.

The protein composition of mitochondria was analyzed by nano-LC-MS and a differential analysis between CFS patient and his healthy brother was performed. Overall, we identified 1007 proteins with a number of peptides used for quantitation ⩾2. Out of 1007 proteins detected using the label-free approach, 194 mitochondrial proteins were significantly modified in CFS. Among these 194 proteins, 41 had a fold variation in CFS with respect to control greater than 2.0, 34 were upregulated in CFS and 7 were downregulated. Table 2 contains data of these 41 proteins. Table containing the remaining 153 proteins is supplied as Supplementary Table S1.

These 41 proteins were included in the analysis with IPA. Each identified protein was converted to its gene and mapped to its corresponding gene object in the IPA knowledge base.

**Figure 1:**
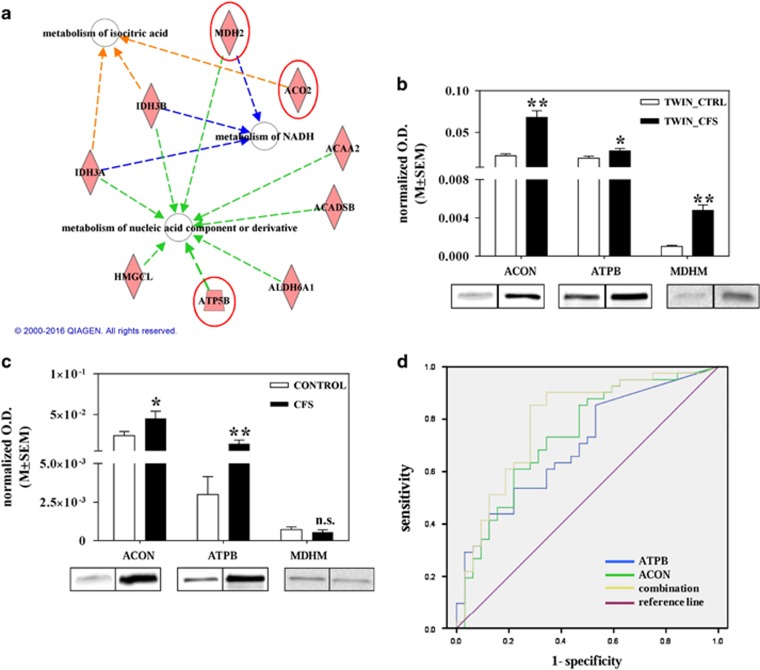
(**a**) Ingenuity Pathway Analysis (IPA). The most statistically significant 41 proteins (*P<*0.05; fold⩾2) were included in the analysis with IPA. The first three top-ranked biological functions were as follows: metabolism of isocitric acid, metabolism of NADH and metabolism of nucleic-acid component or derivative. Selected proteins for validation are indicated with red circles. (**b**) Expression of aconitate hydratase (ACON), ATP synthase subunit beta (ATPB) and malate dehydrogenase (MDHM) in whole saliva (WS) of the twins. Histograms of the normalized optical density (O.D.) obtained in WS of the twins discordant for chronic fatigue syndrome (CFS). Each bar represents the mean±s.e.m.; below, the representative immunoreactive bands are depicted. CTRL, control (*P<0.05, **P<0.01). (**c**) Expression of ACON, ATPB, MDHM in WS of patients and controls. Histograms of the normalized O.D. obtained in WS of patients and healthy controls (CTRL). Each bar represents the mean±s.e.m. of the O.D. obtained from each samples of the two classes of analysis; representative immunoreactive bands are depicted below the histograms (*P<0.05, **P<0.01). (**d**) Receiver operating characteristic (ROC) curves. Receiver operating characteristic curves in case of predicted probability for ATPB, ACON and their combination. ROCs were determined for each marker, comparing healthy subjects and CFS patients.

We utilized IPA to retrieve the known functions of each protein. As one protein may have multiple functions, we selected the functions with the highest statistical significance. The first three top-ranked biological functions were as follows: metabolism of isocitric acid (*P*-value=1.83e^−^^08^), metabolism of NADH (*P*-value=4.00e^−^^07^) and metabolism of nucleic-acid component or derivative (*P*-value=8.15e^−^^07^). These significantly over-represented categories, together with the related molecules found in our study, are depicted in Figures 1a. For the validation we selected a protein for each category by considering both fold variation and the *P*-value of proteins from the proteomics analysis (see Supplementary Results 2 and Supplementary Tables S2).

### Expression of biomarkers in WS

We selected three mitochondrial proteins for the validation on WS—ACON, ATPB and MDHM. First of all, we assessed whether their variation in CFS with respect to control was confirmed in WS of the twin brothers. As depicted in Figure 1b, the upregulation in CFS was corroborated by WB for all the proteins. ACON, ATPB and MDHM showed an increase of 3.1, 1.5 and 4.7, with a significant *P*-value of 0.006, 0.04 and 0.003, respectively (Table 3).

Thereafter, we moved to the analysis of these proteins in a vast cohort of CFS patients (*n*= 45) and healthy subjects (*n*= 45). We started with a global analysis of the expression in WS of these proteins before moving to the examination of different CFS subtypes.

Table 3 and Figure 1c summarize these results. The changes for ACON and ATPB were consistent with the results from nano-LC-MS, but not for MDHM.

**Figure 2:**
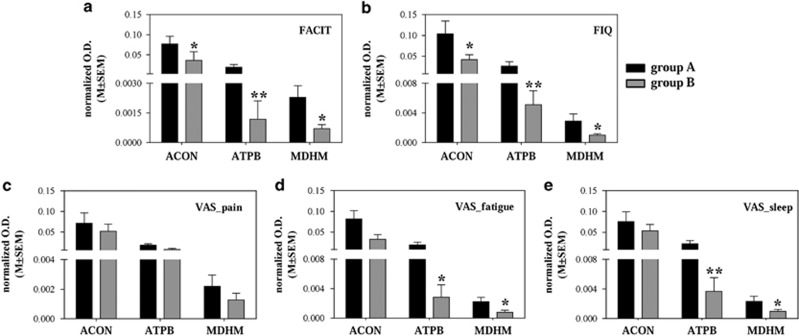
Expression of aconitate hydratase (ACON), ATP synthase subunit beta (ATPB) and malate dehydrogenase (MDHM) in whole saliva (WS) of groups A and B of patients. Chronic fatigue syndrome (CFS) patients were split into two groups considering as reference the twin suffering from CFS. Group A: patients with clinical features whose values were similar or greater than those of the ill twin. Group B: patients with lower clinical values. The histograms report the normalized optical density (O.D.). Each bar represents the mean±s.e.m. (*P<0.05, **P<0.01).

Then, we classified patients according to their clinical features from questionnaires, that is, we examined the following: FACIT, FIQ, VAS_pain, VAS_fatigue and VAS_sleep. CFS patients were split into two groups considering as reference the twin suffering from CFS. One group with the clinical features whose values were in common with (or greater than) those of the ill twin (group A), and the second with the patients with lower values (group B). Intensities were higher for each of the three markers in group A with respect to group B (Figure 2). Details are given in SR3.

### ROC curves

ROC curves were calculated to assess the clinical potential of our selected and validated proteins to distinguish CFS from healthy subjects in WS. The AUCs were calculated for each protein individually, showing whether each marker alone can discriminate CFS from healthy subjects. ATPB and ACON were better differentiating biomarkers than MDHM. ATPB had an AUC of 0.700, with sensitivity=54% and specificity=78%. ACON had an AUC of 0.738, with sensitivity=61% and specificity=78%. Moreover, using a logistic regression analysis, we investigated whether the discriminative power of each marker could be potentially increased with the combination of various markers. With this purpose, we tested all the different combinations in order to select the best association of biomarkers useful to discriminate CFS from healthy subjects. Thus, we found that the discriminative power slightly increased if ATPB was combined with ACON, as shown in Figure 1d. Indeed, the AUC increased to 0.793 and the sensitivity to 85%, but the specificity was 72%. Finally, we also calculated ROC curves for testing the ability of biomarkers to differentiate patients from controls according to the clinical parameters—only FACIT gave good results. When we split patients according to FACIT, for ATPB and ACON we obtained a ROC curve (group A versus controls) with an AUC of 0.780 and 0.722, respectively, whereas the combined ROC curve had an AUC of 0.844 (sensitivity=93% and specificity=70%).

### Clinical correlations

Finally, statistical correlations were carried out calculating the Spearman's rank correlation coefficient. The considered clinical parameters were as follows: age, age of onset, duration of disease and the hematochemical parameters (for example, TNF-α, IL-6 and so on; see the ‘Patients' section). ACON correlated negatively with IL-2, with a Spearman's rank correlation coefficient of −0.458 (*P*-value=0.010). The levels of ATPB correlated positively with TNF-α and IFN-γ. The Spearman's rank correlation coefficient was 0.352 (*P*-value=0.048) and 0.516 (*P*-value=0.004) for TNF-α and IFN-γ, respectively. MDHM also correlates with TNF-α coefficient 0.447, *P*-value=0.013.

## Discussion

The discovery and validation of more objective markers could provide an important support for CFS diagnosis and therapy. As evidence suggests that CFS can be underpinned by mitochondrial dysfunctions, we analyzed mitochondrial proteins obtained from platelets. Platelets were from a couple of monozygotic twins discordant for CFS, which were the subjects of our previous study.^20^ Nano-LC-MS highlighted the changes in expression of 41 proteins with a *P*-value<0.05 and fold of difference greater than 2. Among these proteins, we selected for the validation three proteins from each of the three most significant ‘biological functions' associated with our proteins pointed out after IPA analysis.

These 3 proteins were: ACON for ‘metabolism of isocitric acid', MDHM for ‘metabolism of NADH' and ATPB for the ‘metabolism of nucleic-acid component or derivative'. Moreover, validation of our candidate protein biomarkers was restricted by the availability of antibodies that can allow detection of proteins in saliva samples.

Despite this study analyzed only two subjects, the differences of protein expression could be linked to the disease itself, as the twins differed only regarding the presence of CFS.^20^ By this way, our study on a couple of twins could permit to focus on some biomarkers that can be searched in a wide number of patients in order to confirm their usefulness in CFS diagnosis. We decided to explore the WS of CFS patients preferring to adopt a less invasive procedure in collecting the samples. Although WS is a less complex fluid, it has been proven to be a promising diagnostic tool, which reflects the physiological function of the body.^40, 41^First of all, it was mandatory to check whether the significant increase of these proteins in CFS, with respect to control, could be verified in WS of the twins brothers. Actually, we confirmed the results from nano-LC-MS.

The pathway analysis highlighted the ‘metabolism of NADH' as one of the most important biological functions involved in CFS. The NAD^+^/NADH ratio has an omnipresent role in regulating the intracellular redox status and, therefore, represents a function of the metabolic state. Some studies suggest that NADH concentrations are significantly lower in CFS patients compared with healthy controls.^42, 43, 44^ It is likely that high levels of MDHM and isocitrate dehydrogenase observed in CFS may be an adaptive change to deficiencies of NADH which, in turn, has a critical role in mitochondrial ATP production.

ATPB is the subunit beta of the ATP synthase, the key enzyme for ATP production. In addition to the increase of ATPB in CFS, we also detected significant increases of both subunits alpha and gamma using nano-LC-MS (*P*-value of 0.005 and 0.001 respectively; Supplementary Table S1). Tiredness and energy are tightly connected; therefore, an attractive hypothesis is that there is a metabolic dysfunction with the result that enough energy is not being produced. In this perspective the increase can be explained as an attempt to increase the ATP production. Indeed, there are studies suggesting that the decrease in mitochondrial ATP synthesis in CFS patients is not caused by a defect in the enzyme complexes catalyzing oxidative phosphorylation, but in another factor.^45^ In order for ADP to be recycled, and for ATP to pass into the cytosol from the matrix, the exchange of cytoplasmic ADP with mitochondrial ATP across the mitochondrial inner membrane is needed. This function is provided by a translocator protein. Consistent with other lines of work,^17, 46^ mitochondrial ADP/ATP translocase expression is modified in our study: translocase 2 and 3 decreased in CFS (*P*-value 0.009 and 0.002, respectively; Supplementary Table S1).

Together with ATPB, the most promising biomarker seems to be ACON. This is the mitochondrial form of aconitase, an enzyme that catalyses the stereo-specific isomerization of citrate to isocitrate via cis-aconitate in the tricarboxylic acid cycle. ACON is commonly used as a biomarker for oxidative stress and has been suggested to serve as a sensor of redox status.^47^ Indeed, the reaction between ACON and superoxide generates free hydroxyl radical. It is likely that the reaction between superoxide and ACON may enhance mitochondrial oxidative damage associated with the pathophysiology of CFS.^48^ As a matter of fact, the excessive production of ROS and nitrogen species is usually related to CFS,^16^ and we found a significant upregulation of superoxide dismutase (*P*-value=0.0007; Table 2).

After the validation on the WS of twins, we have broadened the validation to a big cohort of CFS patients. Results for ATPB and ACON corroborated the upregulation in CFS, and their clinical potential to discriminate patients from controls was evaluated. ROC curves highlighted good levels of specificity but low sensitivity. If the biomarkers were combined, we obtained a better performance in diagnosis, and the sensitivity increased to 85%, despite a little decrease in specificity.

On the other hand, the MDHM alteration found in twins was not confirmed in the global cohort of patients, but we could observe its increase when we considered only the patients who had similar or higher clinical parameter values than the twin affected by CFS. This finding brought us to inspect whether other putative biomarkers could also demarcate distinct CFS subgroups, seeing the great variability of clinical features of our patients as demonstrated by Table 1. Therefore, we have amplified our analysis looking closely at clinical data, with the specific aim to investigate how the biomarkers were linked with patients' clinical characteristics. We analyzed the expression of our proteins after splitting patients according to clinical parameters. For each marker, the values were actually higher in the group of patients who had clinical features similar to the ill twin. It seemed that the increase in expression of these proteins was corresponding to a worse patient outcome.

In addition, the combination of ATPB with ACON gave a ROC curve with sensitivity of 93% and specificity of 70%, when we checked patients with values of FACIT greater than equal to those of CFS twin.

It is noteworthy also to point out that CFS patients with comorbid bipolar disorder, and not with other psychiatric comorbidities, showed a significant upregulation of ATPB expression (*P*-value=0.03, data not shown). In our cohort of patients only eight CFS patients had comorbid bipolar disorder (Table 1); anyway, this aspect deserves future investigation, considering that hampered mitochondrial function in bipolar patients has been reviewed.^49, 50^

Finally, we investigated the correlation of our proteins with hematochemical parameters. Cytokine abnormalities are common in CFS, but the findings are mixed and no individual marker is used as an objective biomarker for the diagnosis or management of CFS. It is noteworthy that the increase of ATPB in CFS correlated positively with the increase in IFN-γ and TNF-α. Immunological dysregulation has been always proposed as a significant component of the CFS, and CFS patients had usually high levels of pro-inflammatory cytokines, such as IFN-γ and TNF-α.^14, 51, 52^ In addition, MDHM correlated positively with TNF-α. These correlations are instructive, as all these variables can be thought as markers. It is interesting to point out that ACON levels correlated negatively with IL-2, in line with the fact that the twin with CFS had lower levels of IL-2 with respect to his healthy twin brother.^20^

In conclusion, these results suggest that our potential markers could be one of the criteria to be taken into account for helping in diagnosis. Furthermore, even if these proteins may not necessarily be useful for a universal diagnosis of CFS, given the huge heterogeneity of the illness, they offer a strong potential for characterizing subsets. The identification of biomarkers present in particular subgroups of CFS patients may help in shedding light upon the complex entity of CFS. Moreover, it could help in developing tailored treatments.

By this way, this work may contribute to the continuous development of a more clear framework for a complex and nuanced disease such as CFS.

## Figures and Tables

**Table 1:** Demographics, questionnaire score, hematochemical parameters and psychiatric evaluation of CFS patients

*Demographics*	*CFS twin*	*CFS patients (*n=*45)*	*Mean*±s.d.	*Min–max*
Sex (M/F)	M	22/23		
Age (y)	41		38.6±11.9	19–63
Duration of symptoms (y)	9		7.2±5.6	1–21
Latency of diagnosis (y)	7		5.6±4.6	1–19

*Questionnaire*	*CFS twin*	*Mean±s.d.*	*Min–max*	*Hematochemical parameters*	*CFS twin*	*Mean±s.d.*	Min–max
FIQ	61.2	55±21	11–93	TNF-α	18.1	9.8±4.3	2.1–29
VAS pain	3	3.4±3.1	0–10	IFN-γ	0.2	1.6±3.6	0–19.3
VAS fatigue	8	7.7±2	2–10	IL-6	2	1.8±2.3	0.1–0.8
VAS sleep	8	7.4±2	2–10	IL-10	2	3.6±3.3	0.3–13
FACIT	26	30±10	4–48	IL-2	10	31±38	2–152
SF-36							
Physical functioning	50	59±19	20–100	IL-8	0.3	0.4±0.01	0.2–0.6
Role physical	0	15±25	0–75	IL-1b	0.2	0.7±1.8	0.2–9.9
Bodily pain	84	49±33	0–100	Cortisole	18	19±5.5	10–29.7
General health status	10	36±14	15–82	ACTH	13	32±21	10–84
Vitality	35	24±19	0–70	5-HT	88	180±187	16–887
Social activities	25	32±27	0–87	GH	0	0.6±0.7	0–2.3
Role emotional	66	46±42	0–100	IGF-1	190	339±144	139–607
Mental health	64	61±19	8–84	Vit D3	25	16±7.7	1.5–38

*Psychiatric evaluation*	*CFS twin*	*CFS patients (*n=*45)*	*%*	*Psychiatric comorbidities*
Total	—	21/45	(47%)	
Panic disorder		13/45	(29%)	
			4/13	+ Bipolar disorder II
			1/13	+ Eating disorder
Bipolar disorder II		4/45	(9%)	
Major depression		3/45	(7%)	
Eating disorder		1/45	(2%)	

Abbreviations: ACTH, adrenocorticotropic hormone; CFS, chronic fatigue syndrome; F, female; FACIT, Functional Assessment of Chronic Illness Therapy-Fatigue Scale; FIQ, Fibromyalgia Impact Questionnaire; IGF-1, insulin-like growth factor-1; GH, growth hormone; 5-HT, serotonin; IL, interleukin; M, male; SF-36, short form-36; TNF, tumor necrosis factor; VAS, Visual Analog Score Scale; Vit D3, vitamin D3; y, year.

**Table 2:** List of mitochondrial proteins found differentially expressed by nano-LC-MS analysis, and with a fold of change ⩾2, in mitochondria from platelets of the twins discordant for CFS

*ID*	*Protein*	*Name*	*a*	*CTRL; M*±s.d.	*CFS; M*±s.d.	*b*	*c*
P24752	THIL	Acetyl-CoA acetyltransferase	13	0.57±0.005	1.46±0.007	0.00003	2.5
P00505	AATM	Aspartate aminotransferase	16	3.23±0.05	9.82±0.19	0.0002	3.0
P42765	THIM	3-ketoacyl-CoA thiolase	11	1.54±0.03	6.46±0.13	0.0002	4.2
Q16762	THTR	Thiosulfate sulfurtransferase	14	2.82±0.17	6.55±0.19	0.0002	2.3
Q13011	ECH1	Delta(3,5)-Delta(2,4)-dienoyl-CoA isomerase	12	0.42±0.003	0.95±0.01	0.0002	2.2
P55809	SCOT1	Succinyl-CoA:3-ketoacid coenzyme A transferase 1	20	1.13±0.03	2.41±0.05	0.0002	2.1
P22830	HEMH	Ferrochelatase	4	0.84±0.07	1.72±0.09	0.0003	2.1
Q9NUB1	ACS2L	Acetyl-coenzyme A synthetase 2-like	10	0.90±0.01	2.56±0.04	0.0004	2.8
P06576	ATPB	ATP synthase subunit beta	24	0.32±0.0059	0.71±0.004	0.0004	2.1
O43837	IDH3B	Isocitrate dehydrogenase (NAD) subunit beta	7	2.74±0.04	6.47±0.09	0.001	2.4
P30084	ECHM	Enoyl-CoA hydratase	13	0.93±0.04	2.40±0.06	0.001	2.6
O60488	ACSL4	Long-chain fatty-acid—CoA ligase 4	4	0.14±0.004	0.05±0.003	0.001	0.4
P30038	AL4A1	Delta-1-pyrroline-5-carboxylate dehydrogenase	10	0.90±0.02	2.31±0.04	0.001	2.6
Q9UIJ7	KAD3	GTP:AMP phosphotransferase AK3	10	3.31±0.009	7.42±0.18	0.001	2.2
P04179	SODM	Superoxide dismutase (Mn)	13	2.93±0.007	6.34±0.16	0.001	2.2
P13804	ETFA	Electron transfer flavoprotein subunit alpha	12	0.52±0.02	1.06±0.02	0.001	2.0
Q9Y512	SAM50	Sorting and assembly machinery component 50 homolog	7	0.10±0.002	0.05±0.0005	0.001	0.5
Q5JRX3	PREP	Presequence protease	12	1.82±0.03	5.96±0.23	0.001	3.3
P40926	MDHM	Malate dehydrogenase	17	0.56±0.018	1.30±0.03	0.001	2.3
Q99798	ACON	Aconitate hydratase	35	0.92±0.004	2.01±0.07	0.001	2.2
P35914	HMGCL	Hydroxymethylglutaryl-CoA lyase	9	0.64±0.02	1.44±0.02	0.001	2.3
Q9BX68	HINT2	Histidine triad nucleotide-binding protein 2	4	1.48±0.12	3.35±0.19	0.001	2.3
Q9HAV7	GRPE1	GrpE protein homolog 1	3	0.35±0.02	1.08±0.02	0.001	3.1
P45954	ACDSB	Short/branched chain-specific acyl-CoA dehydrogenase	11	0.32±0.03	0.72±0.05	0.002	2.2
P30405	PPIF	Peptidyl-prolyl *cis–trans* isomerase F	10	0.71±0.009	1.47±0.09	0.005	2.1
P54886	P5CS	Delta-1-pyrroline-5-carboxylate synthase	3	1.52±0.076	4.68±0.3	0.002	3.1
O95563	MPC2	Mitochondrial pyruvate carrier 2	3	0.38±0.008	0.19±0.007	0.002	0.5
P48735	IDHP	Isocitrate dehydrogenase (NADP)	25	0.34±0.008	0.74±0.04	0.002	2.2
Q9H2U2	IPYR2	Inorganic pyrophosphatase 2	5	0.16±0.002	0.36±0.02	0.002	2.3
P30042	ES1	ES1 protein homolog	7	6.10±0.14	14.62±0.71	0.003	2.4
P31937	3HIDH	3-Hydroxyisobutyrate dehydrogenase	4	0.43±0.02	1.14±0.07	0.003	2.7
P50213	IDH3A	Isocitrate dehydrogenase (NAD) subunit alpha	6	1.70±0.06	3.88±0.26	0.003	2.3
O95571	ETHE1	Persulfide dioxygenase ETHE1	4	1.31±0.05	3.19±0.18	0.003	2.4
P11310	ACADM	Medium-chain-specific acyl-CoA dehydrogenase	12	0.49±0.01	1.13±0.06	0.004	2.3
Q02252	MMSA	Methylmalonate-–emialdehyde dehydrogenase (acylating)	8	0.83±0.03	1.75±0.07	0.004	2.1
Q86Y39	NDUAB	NADH dehydrogenase (ubiquinone) 1 alpha subcomplex subunit 11	4	1.13±0.05	0.46±0.03	0.004	0.4
P39210	MPV17	Protein Mpv17	2	0.18±0.006	0.08±0.009	0.002	0.4
Q9Y2R0	COA3	Cytochrome *c* oxidase assembly protein 3 homolog	3	0.40±0.02	0.18±0.002	0.005	0.4
Q9HA77	SYCM	Probable cysteine—tRNA ligase	5	1.39±0.07	3.24±0.36	0.008	2.3
P56556	NDUA6	NADH dehydrogenase (ubiquinone) 1 alpha subcomplex subunit 6	2	0.60±0.06	0.28±0.003	0.011	0.5
Q07021	C1QBP	Complement component 1 Q subcomponent-binding protein	5	0.25±0.02	0.56±0.05	0.020	2.2

Abbreviations: a, peptides used for quantitation; b, *P*-value; c, fold CFS/CTRL; CFS, twin brother affected by chronic fatigue syndrome; CTRL, healthy twin brother; ID, UniProt accession number; M±s.d., mean±s.d. of normalized intensity values; nano-LC-MS, nano-liquid chromatography electrospray ionization mass spectrometry.

**Table 3:** Results of western blot analysis

*Protein*	*TWIN_CTRL*	*TWIN_CFS*	P-*value*	*Fold CFS/ctrl*
ACON	0.022±0.004	0.068±0.014	0.006	3.1
ATPB	0.019±0.004	0.028±0.005	0.04	1.5
MDHM	0.001±0.0002	0.005±0.001	0.003	4.7
	*CTRL*	*CFS*	P*-value*	*Fold CFS/ctrl*
ACON	0.024±0.005	0.045±0.009	0.02	1.9
ATPB	0.003±0.001	0.014±0.005	0.006	4.6
MDHM	0.0007±0.0002	0.001±0.0002	n.s.	−1.3

Abbreviations: ACON, aconitate hydratase; ATPB, ATP synthase subunit beta; CFS, chronic fatigue syndrome; Ctrl, control; MDHM, malate dehydrogenase; n.s., not significant.

Normalized optical density are reported as mean±s.e.m.

## Supplementary Material

Supplementary Methods

Supplementary Results

## References

[bib1] Werker CL, Nijhof SL, van de Putte EM. Clinical Practice: chronic fatigue syndrome. Eur J Pediatr 2013; 172: 1293–1298. 23756916 10.1007/s00431-013-2058-8

[bib2] Yancey JR, Thomas SM. Chronic fatigue syndrome: diagnosis and treatment. Am Fam Physician. 2012; 86: 741–746. 23062157

[bib3] Carruthers BM, van de Sande MI, De Meirleir KL, Klimas NG, Broderick G, Mitchell T et al. Myalgic encephalomyelitis: international consensus criteria. J Intern Med 2011; 270: 327–338. 21777306 10.1111/j.1365-2796.2011.02428.x PMC3427890

[bib4] Johnston S, Brenu EW, Staines D, Marshall-Gradisnik S. The prevalence of chronic fatigue syndrome/myalgic encephalomyelitis: a meta-analysis. Clin Epidemiol 2013; 5: 105–110. 23576883 10.2147/CLEP.S39876 PMC3616604

[bib5] Brurberg KG, Fønhus MS, Larun L, Flottorp S, Malterud K. Case definitions for chronic fatigue syndrome/myalgic encephalomyelitis (CFS/ME): a systematic review. BMJ Open 2014; 4: e003973. 10.1136/bmjopen-2013-003973 PMC3918975 24508851

[bib6] Bakken I, Tveito K, Gunnes N, Ghaderi S, Stoltenberg C, Trogstad L et al. Two age peaks in the incidence of chronic fatigue syndrome/myalgic encephalomyelitis: a population-based registry study from Norway 2008–2012. BMC Med 2014; 12: 167. 25274261 10.1186/s12916-014-0167-5 PMC4189623

[bib7] Pheby D, Sneddon P, Heinrich I. Severe ME/CFS in adults - a report from the CHROME database. Bull IACFS/ME 2009; 17: 143–167.

[bib8] Donegan K, Beau-Lejdstrom R, King B, Seabroke S, Thomson A, Bryan P. Bivalent human papillomavirus vaccine and the risk of fatigue syndromes in girls in the UK. Vaccine 2013; 31: 4961–4967. 24001935 10.1016/j.vaccine.2013.08.024

[bib9] Rosenblum H, Shoenfeld Y, Amital H. The common immunogenic etiology of chronic fatigue syndrome: from infections to vaccines via adjuvants to the ASIA syndrome. Infect Dis Clin North Am 2011; 25: 851–863. 22054760 10.1016/j.idc.2011.07.012

[bib10] Magnus P, Brubakk O, Nyland H, Wold BH, Gjessing HK, Brandt I et al. Vaccination as teenagers against meningococcal disease and the risk of the chronic fatigue syndrome. Vaccine 2009; 27: 23–27. 18984023 10.1016/j.vaccine.2008.10.043

[bib11] Shapiro JS. Does varicella-zoster virus infection of the peripheral ganglia cause chronic fatigue syndrome? Med Hypotheses 2009; 73: 728–734. 19520522 10.1016/j.mehy.2009.04.043

[bib12] Exley C, Swarbrick L, Gherardi RK, Authier FJ. A role for the body burden of aluminium in vaccine-associated macrophagic myofasciitis and chronic fatigue syndrome. Med Hypotheses 2009; 72: 135–139. 19004564 10.1016/j.mehy.2008.09.040

[bib13] Nijs J, Nees A, Paul L, De Kooning M, Ickmans K, Meeus M et al. Altered immune response to exercise in patients with chronic fatigue syndrome/myalgic encephalomyelitis: a systematic literature review. Exerc Immunol Rev 2014; 20: 94–116. 24974723

[bib14] Brenu EW, Huth TK, Hardcastle SL, Fuller K, Kaur M, Johnston S et al. Role of adaptive and innate immune cells in chronic fatigue syndrome/myalgic encephalomyelitis. Int Immunol 2014; 26: 233–242. 24343819 10.1093/intimm/dxt068

[bib15] Armstrong CW, McGregor NR, Butt HL, Gooley PR. Metabolism in chronic fatigue syndrome. Adv Clin Chem 2014; 66: 121–172. 25344988 10.1016/b978-0-12-801401-1.00005-0

[bib16] Morris G, Maes M. Mitochondrial dysfunctions in myalgic encephalomyelitis/chronic fatigue syndrome explained by activated immuno-inflammatory, oxidative and nitrosative stress pathways. Metab Brain Dis 2014; 29: 19–36. 24557875 10.1007/s11011-013-9435-x

[bib17] Booth NE, Myhill S, McLaren-Howard J. Mitochondrial dysfunction and the pathophysiology of Myalgic Encephalomyelitis/Chronic Fatigue Syndrome (ME/CFS). Int J Clin Exp Med 2012; 5: 208–220. 22837795 PMC3403556

[bib18] Myhill S, Booth NE, McLaren-Howard J. Chronic fatigue syndrome and mitochondrial dysfunction. Int J Clin Exp Med 2009; 2: 1–16. 19436827 PMC2680051

[bib19] Gorman GS, Elson JL, Newman J, Payne B, McFarland R, Newton JL et al. Perceived fatigue is highly prevalent and debilitating in patients with mitochondrial disease. Neuromuscul Disord 2015; 25: 563–566. 26031904 10.1016/j.nmd.2015.03.001 PMC4502433

[bib20] Ciregia F, Giusti L, Da Valle Y, Donadio E, Consensi A, Giacomelli C et al. A multidisciplinary approach to study a couple of monozygotic twins discordant for the chronic fatigue syndrome: a focus on potential salivary biomarkers. J Transl Med 2013; 11: 243. 24088505 10.1186/1479-5876-11-243 PMC3850462

[bib21] Garcia-Souza LF, Oliveira MF. Mitochondria: biological roles in platelet physiology and pathology. Int J Biochem Cell Biol 2014; 50: 156–160. 24569121 10.1016/j.biocel.2014.02.015

[bib22] Zharikov S, Shiva S. Platelet mitochondrial function: from regulation of thrombosis to biomarker of disease. Biochem Soc Trans 2013; 41: 118–123. 23356269 10.1042/BST20120327

[bib23] Burkhart JM, Vaudel M, Gambaryan S, Radau S, Walter U, Martens L et al. The first comprehensive and quantitative analysis of human platelet protein composition allows the comparative analysis of structural and functional pathways. Blood 2012; 120: e73–e82. 22869793 10.1182/blood-2012-04-416594

[bib24] Gründler K, Angstwurm M, Hilge R, Baumann P, Annecke T, Crispin A et al. Platelet mitochondrial membrane depolarization reflects disease severity in patients with sepsis and correlates with clinical outcome. Crit Care 2014; 18: R31. 24521521 10.1186/cc13724 PMC4056796

[bib25] Silva AC, Almeida S, Laço M, Duarte AI, Domingues J, Oliveira CR et al. Mitochondrial respiratory chain complex activity and bioenergetic alterations in human platelets derived from pre-symptomatic and symptomatic Huntington's disease carriers. Mitochondrion 2013; 13: 801–809. 23707479 10.1016/j.mito.2013.05.006

[bib26] Carelli V, Ghelli A, Ratta M, Bacchilega E, Sangiorgi S, Mancini R et al. Leber's hereditary optic neuropathy: biochemical effect of 11778/ND4 and 3460/ND1 mutations and correlation with the mitochondrial genotype. Neurology 1997; 48: 1623–1632. 9191778 10.1212/wnl.48.6.1623

[bib27] Mann VM, Cooper JM, Krige D, Daniel SE, Schapira AH, Marsden CD. Brain skeletal muscle and platelet homogenate mitochondrial function in Parkinson's disease. Brain 1992; 115: 333–342. 1606472 10.1093/brain/115.2.333

[bib28] Tang WH, Stitham J, Jin Y, Liu R, Lee SH, Du J et al. Aldose reductase-mediated phosphorylation of p53 leads to mitochondrial dysfunction and damage in diabetic platelets. Circulation 2014; 129: 1598–1609. 24474649 10.1161/CIRCULATIONAHA.113.005224 PMC3989377

[bib29] Avila C, Huang RJ, Stevens MV, Aponte AM, Tripodi D, Kim KY et al. Platelet mitochondrial dysfunction is evident in type 2 diabetes in association with modifications of mitochondrial anti-oxidant stress proteins. Exp Clin Endocrinol Diabetes 2012; 120: 248–251. 21922457 10.1055/s-0031-1285833 PMC6122851

[bib30] Fukuda K, Straus SE, Hickie I, Sharpe MC, Dobbins JG, Komaroff A. The chronic fatigue syndrome: a comprehensive approach to its definition and study. International Chronic Fatigue Syndrome Study Group. Ann Intern Med 1994; 121: 953–959. 7978722 10.7326/0003-4819-121-12-199412150-00009

[bib31] First MB, Spitzer RL, Gibbon M, JBW Williams. Structured Clinical Interview for DSM-IV Axis I Disorders, Research Version, Patient Edition. (SCID-I/P). Biometrics Research, New York State Psychiatric Institute: New York, NY, USA, 2002.

[bib32] Giusti L, Baldini C, Bazzichi L, Ciregia F, Tonazzini I, Mascia G et al. Proteome analysis of whole saliva: a new tool for rheumatic diseases-the example of Sjögren's syndrome. Proteomics 2007; 7: 1634–1643. 17436266 10.1002/pmic.200600783

[bib33] Manza LL, Stamer SL, Ham AJ, Codreanu SG, Liebler DC. Sample preparation and digestion for proteomic analyses using spin filters. Proteomics 2005; 5: 1742–1745. 15761957 10.1002/pmic.200401063

[bib34] Wiśniewski JR, Zougman A, Nagaraj N, Mann M. Universal sample preparation method for proteome analysis. Nat Method 2009; 6: 359–362. 10.1038/nmeth.1322 19377485

[bib35] Kollipara L, Zahedi RP. Protein carbamylation: *in vivo* modification or *in vitro* artefact? Proteomics 2013; 13: 941–944. 23335428 10.1002/pmic.201200452

[bib36] Burkhart JM, Schumbrutzki C, Wortelkamp S, Sickmann A, Zahedi RP. Systematic and quantitative comparison of digest efficiency and specificity reveals the impact of trypsin quality on MS-based proteomics. J Proteomics 2012; 75: 1454–1462. 22166745 10.1016/j.jprot.2011.11.016

[bib37] Olsen JV, de Godoy LM, Li G, Macek B, Mortensen P, Pesch R et al. Parts per Million Mass Accuracy on an Orbitrap Mass Spectrometer via Lock Mass Injection into a C-trap. Mol Cell Proteomics 2005; 4: 2010–2021. 16249172 10.1074/mcp.T500030-MCP200

[bib38] Vaudel M, Burkhart JM, Zahedi RP, Oveland E, Berven FS, Sickmann A et al. PeptideShaker enables reanalysis of MS-derived proteomics data sets. Nat Biotechnol 2015; 33: 22–24. 25574629 10.1038/nbt.3109

[bib39] Giusti L, Mantua V, Da Valle Y, Ciregia F, Ventroni T, Orsolini G et al. Search for peripheral biomarkers in patients affected by acutely psychotic bipolar disorder: a proteomic approach. Mol Biosyst 2014; 10: 1246–1254. 24554194 10.1039/c4mb00068d

[bib40] Lima DP, Diniz DG, Moimaz SAS, Sumida DH, Okamoto AC. Saliva: reflection of the body. Intl J Infect Dis 2010; 14: e184–e188. 10.1016/j.ijid.2009.04.022 19726214

[bib41] Javaid MA, Ahmed AS, Durand R, Tran SD. Saliva as a diagnostic tool for oral and systemic diseases. J Oral Biol Craniofac Res 2016; 6: 66–75. 26937373 10.1016/j.jobcr.2015.08.006 PMC4756071

[bib42] Mikirova N, Casciari J, Hunninghake R. The assessment of the energy metabolism in patients with chronic fatigue syndrome by serum fluorescence emission. Altern Ther Health Med 2012; 18: 36–40. 22516851

[bib43] Castro-Marrero J, Cordero MD, Segundo MJ, Sáez-Francàs N, Calvo N, Román-Malo L et al. Does oral coenzyme Q10 plus NADH supplementation improve fatigue and biochemical parameters in chronic fatigue syndrome? Antioxid Redox Signal 2015; 22: 679–685. 25386668 10.1089/ars.2014.6181 PMC4346380

[bib44] Castro-Marrero J, Sáez-Francàs N, Segundo MJ, Calvo N, Faro M, Aliste L et al. Effect of coenzyme Q10 plus nicotinamide adenine dinucleotide supplementation on maximum heart rate after exercise testing in chronic fatigue syndrome - a randomized, controlled, double-blind trial. Clin Nutr 2015; 35: 826–834. 26212172 10.1016/j.clnu.2015.07.010

[bib45] Vermeulen RC, Kurk RM, Visser FC, Sluiter W, Scholte HR. Patients with chronic fatigue syndrome performed worse than controls in a controlled repeated exercise study despite a normal oxidative phosphorylation capacity. J Transl Med 2010; 8: 93. 20937116 10.1186/1479-5876-8-93 PMC2964609

[bib46] Hsiao CP, Wang D, Kaushal A, Saligan L. Mitochondria-related gene expression changes are associated with fatigue in patients with nonmetastatic prostate cancer receiving external beam radiation therapy. Cancer Nurs 2013; 36: 189–197. 23047795 10.1097/NCC.0b013e318263f514 PMC4665987

[bib47] Bubber P, Hartounian V, Gibson GE, Blass JP. Abnormalities in the tricarboxylic acid (tca) cycle in brain of schizophrenia patients. Eur Neuropsychopharmacol 2011; 21: 254–260. 21123035 10.1016/j.euroneuro.2010.10.007 PMC3033969

[bib48] Vasquez-Vivar J, Kalyanaraman B, Kennedy MC. Mitochondrial aconitase is a source of hydroxyl radical. An electron spin resonance investigation. J Biol Chem 2000; 275: 14064–14069. 10799480 10.1074/jbc.275.19.14064

[bib49] Shao L, Martin MV, Watson SJ, Schatzberg A, Akil H, Myers RM et al. Mitochondrial involvement in psychiatric disorders. Ann Med 2008; 40: 281–295. 18428021 10.1080/07853890801923753 PMC3098560

[bib50] Callaly E, Walder K, Morris G, Maes M, Debnath M, Berk M. Mitochondrial dysfunction in the pathophysiology of bipolar disorder: effects of pharmacotherapy. Mini Rev Med Chem 2015; 15: 355–365. 25807948 10.2174/1389557515666150324122026

[bib51] Brenu EW, van Driel ML, Staines DR, Ashton KJ, Ramos SB, Keane J et al. Immunological abnormalities as potential biomarkers in chronic fatigue syndrome/myalgic encephalomyelitis. J Transl Med 2011; 9: 81. 21619669 10.1186/1479-5876-9-81 PMC3120691

[bib52] Klimas NG, Broderick G, Fletcher MA. Biomarkers for chronic fatigue. Brain Behav Immun 2012; 26: 1202–1210. 22732129 10.1016/j.bbi.2012.06.006 PMC5373648

