# Supplementary material for: Bottom-up proteomics suggests an association between differential expression of mitochondrial proteins and chronic fatigue syndrome
Source: Transl Psychiatry. 2016 Sep 27;6(9):e904–. doi: 10.1038/tp.2016.184 (PMC5048217; doi:10.1038/tp.2016.184)
Supplement: Supplementary Methods [file tp2016184x1.doc]

**SM1 Supplementary Methods 1_Subjects**

Forty-five patients affected by CFS were consecutively recruited and satisfied the inclusion and exclusion criteria here listed. Inclusion Criteria: Diagnosis of CFS according to the classification criteria of Fukuda of 1994; Aged between 18 and 65; Patients of both sexes; Acceptance of the protocol and signed informed consent.

Exclusion Criteria: Patients aged > 65 and < 18; Status of alleged or established pregnancy and lactation; Lack of consent by the patient; Presence of active rheumatic diseases, infectious and / or unstable medical condition; Withdrawal of informed consent by the patient.

Forty-five healthy subjects were included as controls. Control subjects, in addition to satisfy the inclusion and exclusion criteria listed below, were identified among those who voluntarily donated blood at the blood transfusion center, prior informed consent. They had normal haematochemical parameters.

Inclusion Criteria: Healthy constitution; Aged between 18 and 65; Subjects of both sexes; Acceptance of the protocol and signed informed consent.

Exclusion criteria: patients aged > 65 and < 18; Status of alleged or established pregnancy and lactation; Presence of active rheumatic diseases, psychiatric disorders, infectious and/or unstable medical condition; Lack of consent by the subject; Withdrawal of informed consent by the subject.

**SM2 Supplementary Methods 2_Evaluation of the mitochondrial preparation**

Western Blotting analysis was performed to evaluate the purity of the mitochondria. Ten µg of proteins were resolved by 12% SDS-PAGE gels and transferred onto nitrocellulose membranes (0.2 µm). Non-specific binding was prevented by blocking membranes with 3% low fat dried milk, 0.2% (v/v) Tween 20 in PBS (10 mM NaH_2_PO_4_, pH 7.4, 0.9% NaCl) (PBS/milk) for 1 h at room temperature. After blocking, membranes were incubated 2 h at room temperature in PBS/milk with primary antibody which was composed by a cocktail of 4 different antibodies each targeting a specific cellular marker (Abcam, UK), the dilution was 1:250. A HRP-conjugated goat antimouse (1:10,000 dilution; PerkinElmer, MA, USA) was used as a secondary antibody. Immunoblots were developed using the ECL detection system (PerkinElmer, MA, USA). The chemiluminescent images were acquired by LAS4010 (GE Health Care, WI, USA).

The integrity of mitochondria was assayed by measuring the activity of cytochrome oxidase in 10 µg of mitochondrial preparation (Cytochrome Oxidase Activity Colorimetric Assay Kit; BioVision, CA, USA). The activity of the enzyme is determined by following the oxidation of reduced Cytochrome c as an adsorbance decrease at 550 nm, in the presence of 1 mM *n*-dodecyl β-D-maltoside.

**SM3 Supplementary Methods 3_ Signaling pathway analysis**

Proteins differentially expressed were functionally analyzed through the use of QIAGEN’s IPA (Ingenuity® System Inc., QIAGEN Redwood City, CA, USA) to select candidates for validation.

A dataset containing Uniprot accession numbers, along with corresponding p-value and comparison ratios was uploaded into the software. Each identified protein was converted to its gene and mapped to its corresponding gene object in the IPA knowledge base. The ''Core Analysis' function, included in IPA, helps to interpret the data in the context of biological processes, pathways and networks. After the analysis, IPA provides details of the most significant biological function, associated with our proteins, that are ordered by statistical significance. The software shows which proteins are associated to the most significant functions, and how this functions are related.
