# Supplementary material for: Bottom-up proteomics suggests an association between differential expression of mitochondrial proteins and chronic fatigue syndrome
Source: Transl Psychiatry. 2016 Sep 27;6(9):e904–. doi: 10.1038/tp.2016.184 (PMC5048217; doi:10.1038/tp.2016.184)
Supplement: Supplementary Results [file tp2016184x2.doc]

**SR1 Supplementary Results 1_ Evaluation of mitochondrial enrichment**

To evaluate the quality of the isolated mitochondria, enzymatic assay, and WB analysis were employed. The cytochrome oxidase assay showed the decrease of reduced Cytochrome c revealing the presence of mitochondrial activity. The rate of the reaction has been calculated in the linear range (2 minutes) after the addition of *n*-dodecyl β-D-maltoside and specific activity was 0.93 units/mg.

In addition, 4 different cellular markers were tested by WB analysis: Anti-Sodium Potassium ATPase antibody (Plasma Membrane Marker), Anti-ATP5A antibody (Mitochondrial Marker), Anti-GAPDH antibody (Cytosolic Marker), and Anti-Histone H3 (Nuclear Marker). As shown in figure S1, the mitochondrial marker was effectively enriched in the mitochondrial fraction and undetectable in the cytosolic fraction. The nuclear marker was absent, since platelets do not have nucleus, but, in addition, this means that we do not have contamination by other blood cells. While the sarcolemmal marker was basically reduced, the cytosolic marker was slightly present in the mitochondrial fraction.

The mitochondria enrichment is very challenging because these organelles have close interaction with other cellular elements. Therefore, even if mitochondria have been well fractionated, proteins contaminants could not be completely excluded from the samples. Therefore, WB data (figure S1) and cytochrome oxidase assay confirmed that we reached satisfactory mitochondria enrichment.

**SR2 Supplementary Results 2_ Signaling pathway analysis**

Table S2 lists the most significant “biological functions” associated with our proteins, with the list of corresponding genes involved, obtained from Signaling pathway analysis with IPA. For the validation, we selected a protein for each of the first three top ranked biological functions. The proteins were selected by considering fold variation and the p-value. In table S3 we listed all the proteins belonging to the 3 most significant “biological functions”. Identified proteins have been ranked by p-value to highlight that our selected proteins are among the most significant in each category. Moreover, the validation was restricted by the availability of antibodies which can allow detection of proteins in saliva samples. When tested, not all the antibodies gave immunoreactive signal on WS, or a good signal. This had further limited the selection of markers.

**SR3 Supplementary Results 3_ Expression of biomarkers in WS**

Moreover, we observed a statistically significant increment for the 3 proteins when considering FACIT and FIQ. Indeed, ACON, ATPB and MDHM showed p-values of 0.04, 0.0047 and 0.017, respectively, when considering FACIT. In regard to FIQ, p-values were 0.037, 0.0034, and 0.0236 for ACON, ATPB and MDHM respectively. ATPB and MDHM had, respectively, p-values of 0.016 and 0.024 considering VAS_fatigue; while 0.0091, and 0.04 where their p-values for VAS_sleep (figure 2).

**Supplementary Figure_Legend figure S1**

Determination of the purity of the mitochondrial preparation. Western blot analysis of mitochondrial fraction extracted from platelets. PM: Anti-Sodium Potassium ATPase antibody–Plasma Membrane Marker; theoretical molecular weight (th MW), 113 kDa. MITO: Anti-ATP5A antibody–Mitochondrial Marker; th MW = 60 kDa. CYTO: Anti-GAPDH antibody–Cytosolic Marker; th MW 36 kDa. MW st: molecular weights of standard proteins.
