# Supplementary figures and images for: Bottom-up proteomics suggests an association between differential expression of mitochondrial proteins and chronic fatigue syndrome
Source: Transl Psychiatry. 2016 Sep 27;6(9):e904–. doi: 10.1038/tp.2016.184 (PMC5048217; doi:10.1038/tp.2016.184)

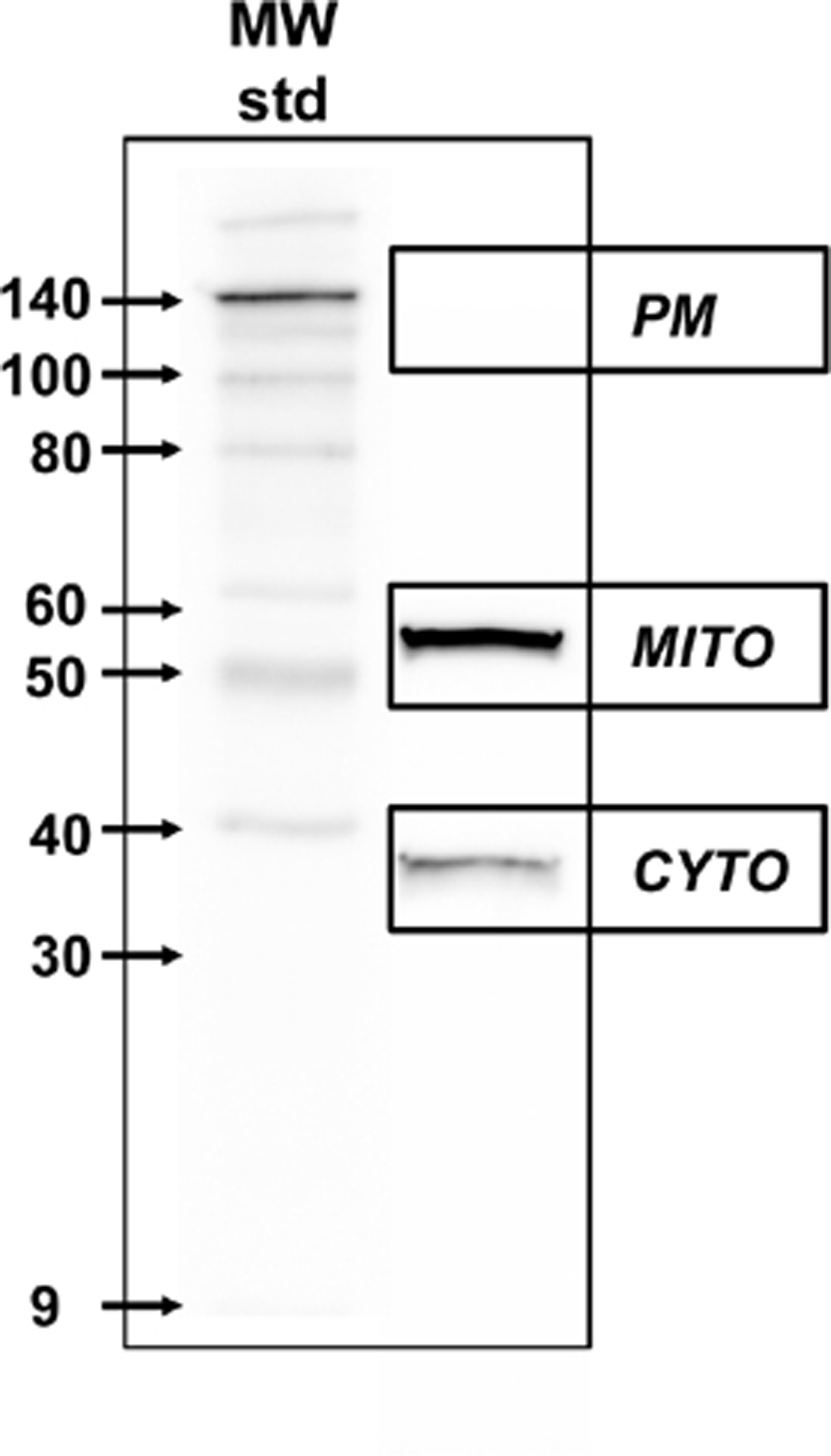

Supplement: Supplementary Figure [file tp2016184x3.tif]
