# Supplementary material for: Bottom-up proteomics suggests an association between differential expression of mitochondrial proteins and chronic fatigue syndrome
Source: Transl Psychiatry. 2016 Sep 27;6(9):e904–. doi: 10.1038/tp.2016.184 (PMC5048217; doi:10.1038/tp.2016.184)
Supplement: Supplementary Table S1 [file tp2016184x4.doc]

**Table S1**. **List of proteins found differentially expressed by nano-LC-MS/MS analysis, with a fold of change < 2. The normalized intensity values are indicated.**

| **ID** | **Protein** | **Name** | **a** | **Healthy** | **CFS** | **b** | **c** |
| --- | --- | --- | --- | --- | --- | --- | --- |
| P19367 | HXK1 | Hexokinase-1 | 42 | 0.40 | 0.44 | 0.00002 | 1.09 |
| P34897 | GLYM | Serine hydroxymethyltransferase, mitochondrial | 12 | 0.66 | 1.14 | 0.00004 | 1.72 |
| O95167 | NDUA3 | NADH dehydrogenase [ubiquinone] 1 alpha subcomplex subunit 3 | 3 | 0.05 | 0.05 | 0.00006 | 1.10 |
| O95299 | NDUAA | NADH dehydrogenase [ubiquinone] 1 alpha subcomplex subunit 10, mitochondrial | 10 | 0.18 | 0.13 | 0.0001 | 0.76 |
| P42704 | LPPRC | Leucine-rich PPR motif-containing protein, mitochondrial | 25 | 0.34 | 0.56 | 0.0001 | 1.64 |
| Q99623 | PHB2 | Prohibitin-2 | 14 | 0.20 | 0.15 | 0.0001 | 0.74 |
| Q9NX63 | CHCH3 | Coiled-coil-helix-coiled-coil-helix domain-containing protein 3, mitochondrial | 9 | 0.23 | 0.13 | 0.0002 | 0.58 |
| Q04837 | SSBP | Single-stranded DNA-binding protein, mitochondrial | 9 | 0.98 | 1.84 | 0.0003 | 1.88 |
| O95139 | NDUB6 | NADH dehydrogenase [ubiquinone] 1 beta subcomplex subunit 6 | 3 | 0.12 | 0.08 | 0.0004 | 0.65 |
| P08574 | CY1 | Cytochrome c1, heme protein, mitochondrial | 8 | 0.15 | 0.08 | 0.0004 | 0.58 |
| O95831 | AIFM1 | Apoptosis-inducing factor 1, mitochondrial | 20 | 0.19 | 0.15 | 0.0004 | 0.78 |
| Q9Y277 | VDAC3 | Voltage-dependent anion-selective channel protein 3 | 15 | 0.14 | 0.10 | 0.0004 | 0.68 |
| Q9P2R7 | SUCB1 | Succinyl-CoA ligase [ADP-forming] subunit beta, mitochondrial | 13 | 2.10 | 3.48 | 0.0005 | 1.66 |
| Q9NSE4 | SYIM | Isoleucine--tRNA ligase, mitochondrial | 13 | 0.84 | 1.28 | 0.0005 | 1.52 |
| O95298 | NDUC2 | NADH dehydrogenase [ubiquinone] 1 subunit C2 | 2 | 0.27 | 0.15 | 0.0006 | 0.57 |
| P11177 | ODPB | Pyruvate dehydrogenase E1 component subunit beta, mitochondrial | 9 | 0.25 | 0.41 | 0.0006 | 1.66 |
| P07954 | FUMH | Fumarate hydratase, mitochondrial | 14 | 0.64 | 0.95 | 0.0006 | 1.47 |
| Q16698 | DECR | 2,4-dienoyl-CoA reductase, mitochondrial | 10 | 0.40 | 0.71 | 0.0006 | 1.79 |
| Q15118 | PDK1 | [Pyruvate dehydrogenase | 12 | 0.49 | 0.75 | 0.0006 | 1.54 |
| O75390 | CISY | Citrate synthase, mitochondrial | 11 | 0.49 | 0.96 | 0.0006 | 1.95 |
| P99999 | CYC | Cytochrome c | 11 | 5.76 | 4.32 | 0.0007 | 0.75 |
| Q16891 | IMMT | Mitochondrial inner membrane protein | 30 | 0.15 | 0.12 | 0.0009 | 0.77 |
| Q9Y6N5 | SQRD | Sulfide:quinone oxidoreductase, mitochondrial | 17 | 0.25 | 0.23 | 0.0010 | 0.91 |
| P17568 | NDUB7 | NADH dehydrogenase [ubiquinone] 1 beta subcomplex subunit 7 | 6 | 0.32 | 0.23 | 0.001 | 0.72 |
| P62072 | TIM10 | Mitochondrial import inner membrane translocase subunit Tim10 | 3 | 1.18 | 2.15 | 0.001 | 1.83 |
| O96000 | NDUBA | NADH dehydrogenase [ubiquinone] 1 beta subcomplex subunit 10 | 9 | 0.27 | 0.16 | 0.001 | 0.58 |
| Q9UKU7 | ACAD8 | Isobutyryl-CoA dehydrogenase, mitochondrial | 9 | 0.74 | 1.10 | 0.001 | 1.49 |
| P23786 | CPT2 | Carnitine O-palmitoyltransferase 2, mitochondrial | 13 | 0.45 | 0.73 | 0.001 | 1.60 |
| O94925 | GLSK | Glutaminase kidney isoform, mitochondrial | 7 | 0.33 | 0.55 | 0.001 | 1.68 |
| P31930 | QCR1 | Cytochrome b-c1 complex subunit 1, mitochondrial | 17 | 0.09 | 0.07 | 0.001 | 0.76 |
| Q99714 | HCD2 | 3-hydroxyacyl-CoA dehydrogenase type-2 | 11 | 0.28 | 0.52 | 0.001 | 1.84 |
| P09622 | DLDH | Dihydrolipoyl dehydrogenase, mitochondrial | 13 | 0.21 | 0.34 | 0.001 | 1.61 |
| P50416 | CPT1A | Carnitine O-palmitoyltransferase 1, liver isoform | 15 | 0.11 | 0.08 | 0.001 | 0.69 |
| P51649 | SSDH | Succinate-semialdehyde dehydrogenase, mitochondrial | 4 | 0.39 | 0.73 | 0.001 | 1.86 |
| P10606 | COX5B | Cytochrome c oxidase subunit 5B, mitochondrial | 6 | 0.04 | 0.03 | 0.001 | 0.76 |
| P55084 | ECHB | Trifunctional enzyme subunit beta, mitochondrial | 25 | 0.23 | 0.27 | 0.001 | 1.18 |
| P36542 | ATPG | ATP synthase subunit gamma, mitochondrial | 10 | 0.19 | 0.21 | 0.002 | 1.08 |
| P22307 | NLTP | Non-specific lipid-transfer protein | 4 | 0.24 | 0.33 | 0.002 | 1.38 |
| P30048 | PRDX3 | Thioredoxin-dependent peroxide reductase, mitochondrial | 6 | 0.47 | 0.82 | 0.002 | 1.72 |
| Q9NVI7 | ATD3A | ATPase family AAA domain-containing protein 3A | 11 | 0.11 | 0.08 | 0.002 | 0.71 |
| Q6UXV4 | APOOL | Apolipoprotein O-like | 9 | 0.35 | 0.20 | 0.002 | 0.57 |
| P00367 | DHE3 | Glutamate dehydrogenase 1, mitochondrial | 23 | 0.21 | 0.32 | 0.002 | 1.53 |
| P27338 | AOFB | Amine oxidase [flavin-containing] B | 20 | 0.14 | 0.09 | 0.002 | 0.66 |
| P14406 | CX7A2 | Cytochrome c oxidase subunit 7A2, mitochondrial | 3 | 0.12 | 0.18 | 0.002 | 1.56 |
| Q9Y4W6 | AFG32 | AFG3-like protein 2 | 17 | 0.16 | 0.11 | 0.002 | 0.73 |
| P30536 | TSPOA | Translocator protein | 3 | 0.38 | 0.23 | 0.002 | 0.62 |
| P24539 | AT5F1 | ATP synthase F | 8 | 0.11 | 0.08 | 0.002 | 0.72 |
| Q3SXM5 | HSDL1 | Inactive hydroxysteroid dehydrogenase-like protein 1 | 7 | 0.15 | 0.10 | 0.002 | 0.69 |
| Q13423 | NNTM | NAD | 38 | 0.17 | 0.12 | 0.002 | 0.73 |
| Q02978 | M2OM | Mitochondrial 2-oxoglutarate/malate carrier protein | 11 | 0.06 | 0.04 | 0.002 | 0.79 |
| O94826 | TOM70 | Mitochondrial import receptor subunit TOM70 | 12 | 0.17 | 0.10 | 0.002 | 0.62 |
| P38117 | ETFB | Electron transfer flavoprotein subunit beta | 12 | 0.44 | 0.62 | 0.002 | 1.41 |
| P12236 | ADT3 | ADP/ATP translocase 3 | 6 | 0.12 | 0.09 | 0.002 | 0.80 |
| Q9P0J0 | NDUAD | NADH dehydrogenase [ubiquinone] 1 alpha subcomplex subunit 13 | 7 | 0.21 | 0.13 | 0.003 | 0.63 |
| O95182 | NDUA7 | NADH dehydrogenase [ubiquinone] 1 alpha subcomplex subunit 7 | 6 | 0.20 | 0.13 | 0.003 | 0.68 |
| P38646 | GRP75 | Stress-70 protein, mitochondrial | 34 | 0.24 | 0.36 | 0.003 | 1.52 |
| P14927 | QCR7 | Cytochrome b-c1 complex subunit 7 | 8 | 0.13 | 0.10 | 0.003 | 0.76 |
| P36776 | LONM | Lon protease homolog, mitochondrial | 14 | 0.25 | 0.42 | 0.003 | 1.69 |
| P43304 | GPDM | Glycerol-3-phosphate dehydrogenase, mitochondrial | 35 | 0.16 | 0.12 | 0.003 | 0.75 |
| P22695 | QCR2 | Cytochrome b-c1 complex subunit 2, mitochondrial | 15 | 0.17 | 0.13 | 0.003 | 0.73 |
| Q9UI09 | NDUAC | NADH dehydrogenase [ubiquinone] 1 alpha subcomplex subunit 12 | 5 | 0.62 | 0.70 | 0.003 | 1.12 |
| O75306 | NDUS2 | NADH dehydrogenase [ubiquinone] iron-sulfur protein 2, mitochondrial | 8 | 0.16 | 0.12 | 0.003 | 0.75 |
| Q96RQ3 | MCCA | Methylcrotonoyl-CoA carboxylase subunit alpha, mitochondrial | 3 | 0.12 | 0.19 | 0.003 | 1.61 |
| P08559 | ODPA | Pyruvate dehydrogenase E1 component subunit alpha, somatic form, mitochondrial | 11 | 0.34 | 0.54 | 0.003 | 1.58 |
| Q16822 | PCKGM | Phosphoenolpyruvate carboxykinase [GTP], mitochondrial | 17 | 0.69 | 1.18 | 0.003 | 1.70 |
| Q15120 | PDK3 | [Pyruvate dehydrogenase | 7 | 0.71 | 1.23 | 0.003 | 1.73 |
| P13073 | COX41 | Cytochrome c oxidase subunit 4 isoform 1, mitochondrial | 12 | 0.18 | 0.15 | 0.003 | 0.83 |
| P40939 | ECHA | Trifunctional enzyme subunit alpha, mitochondrial | 32 | 0.19 | 0.24 | 0.003 | 1.26 |
| P09669 | COX6C | Cytochrome c oxidase subunit 6C | 7 | 0.25 | 0.15 | 0.003 | 0.59 |
| Q9Y6M9 | NDUB9 | NADH dehydrogenase [ubiquinone] 1 beta subcomplex subunit 9 | 7 | 0.11 | 0.07 | 0.003 | 0.67 |
| Q16795 | NDUA9 | NADH dehydrogenase [ubiquinone] 1 alpha subcomplex subunit 9, mitochondrial | 14 | 0.21 | 0.15 | 0.004 | 0.71 |
| Q16836 | HCDH | Hydroxyacyl-coenzyme A dehydrogenase, mitochondrial | 14 | 1.76 | 3.41 | 0.004 | 1.93 |
| P10809 | CH60 | 60 kDa heat shock protein, mitochondrial | 33 | 0.22 | 0.30 | 0.004 | 1.38 |
| P49748 | ACADV | Very long-chain specific acyl-CoA dehydrogenase, mitochondrial | 25 | 0.32 | 0.52 | 0.004 | 1.62 |
| Q9UHQ9 | NB5R1 | NADH-cytochrome b5 reductase 1 | 14 | 0.27 | 0.23 | 0.004 | 0.84 |
| P20674 | COX5A | Cytochrome c oxidase subunit 5A, mitochondrial | 9 | 0.13 | 0.10 | 0.004 | 0.75 |
| O75380 | NDUS6 | NADH dehydrogenase [ubiquinone] iron-sulfur protein 6, mitochondrial | 6 | 0.23 | 0.19 | 0.004 | 0.83 |
| Q00059 | TFAM | Transcription factor A, mitochondrial | 7 | 0.69 | 1.21 | 0.004 | 1.76 |
| Q00325 | MPCP | Phosphate carrier protein, mitochondrial | 13 | 0.16 | 0.11 | 0.004 | 0.69 |
| Q02218 | ODO1 | 2-oxoglutarate dehydrogenase, mitochondrial | 27 | 0.18 | 0.24 | 0.004 | 1.34 |
| O60313 | OPA1 | Dynamin-like 120 kDa protein, mitochondrial | 24 | 0.11 | 0.09 | 0.004 | 0.74 |
| O00483 | NDUA4 | NADH dehydrogenase [ubiquinone] 1 alpha subcomplex subunit 4 | 2 | 0.53 | 0.45 | 0.005 | 0.83 |
| Q9HCC0 | MCCB | Methylcrotonoyl-CoA carboxylase beta chain, mitochondrial | 11 | 0.46 | 0.63 | 0.005 | 1.35 |
| P25705 | ATPA | ATP synthase subunit alpha, mitochondrial | 35 | 0.34 | 0.49 | 0.005 | 1.42 |
| P50440 | GATM | Glycine amidinotransferase, mitochondrial | 9 | 1.99 | 3.81 | 0.005 | 1.92 |
| Q8NE86 | MCU | Calcium uniporter protein, mitochondrial | 10 | 0.13 | 0.09 | 0.005 | 0.67 |
| P23368 | MAOM | NAD-dependent malic enzyme, mitochondrial | 16 | 0.39 | 0.72 | 0.006 | 1.86 |
| P14854 | CX6B1 | Cytochrome c oxidase subunit 6B1 | 4 | 0.13 | 0.11 | 0.006 | 0.82 |
| O95674 | CDS2 | Phosphatidate cytidylyltransferase 2 | 3 | 0.02 | 0.03 | 0.006 | 1.39 |
| Q9UJZ1 | STML2 | Stomatin-like protein 2, mitochondrial | 13 | 0.13 | 0.10 | 0.006 | 0.73 |
| P36957 | ODO2 | Dihydrolipoyllysine-residue succinyltransferase component of 2-oxoglutarate dehydrogenase complex, mitochondrial | 13 | 0.25 | 0.33 | 0.006 | 1.35 |
| Q96IX5 | USMG5 | Up-regulated during skeletal muscle growth protein 5 | 3 | 0.17 | 0.12 | 0.006 | 0.70 |
| O00217 | NDUS8 | NADH dehydrogenase [ubiquinone] iron-sulfur protein 8, mitochondrial | 3 | 0.10 | 0.09 | 0.007 | 0.87 |
| O43181 | NDUS4 | NADH dehydrogenase [ubiquinone] iron-sulfur protein 4, mitochondrial | 3 | 0.05 | 0.05 | 0.007 | 0.92 |
| P61604 | CH10 | 10 kDa heat shock protein, mitochondrial | 8 | 0.97 | 1.58 | 0.007 | 1.62 |
| P36551 | HEM6 | Coproporphyrinogen-III oxidase, mitochondrial | 6 | 0.75 | 0.47 | 0.007 | 0.63 |
| Q6NUK1 | SCMC1 | Calcium-binding mitochondrial carrier protein SCaMC-1 | 9 | 0.17 | 0.11 | 0.008 | 0.67 |
| Q9NX14 | NDUBB | NADH dehydrogenase [ubiquinone] 1 beta subcomplex subunit 11, mitochondrial | 3 | 0.05 | 0.04 | 0.008 | 0.79 |
| O14949 | QCR8 | Cytochrome b-c1 complex subunit 8 | 2 | 0.30 | 0.16 | 0.009 | 0.54 |
| P05141 | ADT2 | ADP/ATP translocase 2 | 5 | 0.14 | 0.09 | 0.009 | 0.65 |
| P48047 | ATPO | ATP synthase subunit O, mitochondrial | 8 | 0.14 | 0.12 | 0.009 | 0.87 |
| Q13637 | RAB32 | Ras-related protein Rab-32 | 8 | 0.03 | 0.03 | 0.009 | 0.79 |
| P26440 | IVD | Isovaleryl-CoA dehydrogenase, mitochondrial | 5 | 0.82 | 1.16 | 0.010 | 1.41 |
| O75947 | ATP5H | ATP synthase subunit d, mitochondrial | 13 | 0.19 | 0.14 | 0.010 | 0.75 |
| Q8NBN7 | RDH13 | Retinol dehydrogenase 13 | 2 | 0.38 | 0.21 | 0.010 | 0.55 |
| P03928 | ATP8 | ATP synthase protein 8 | 2 | 0.08 | 0.05 | 0.011 | 0.64 |
| Q9Y6C9 | MTCH2 | Mitochondrial carrier homolog 2 | 4 | 0.13 | 0.08 | 0.012 | 0.65 |
| Q9NVH1 | DJC11 | DnaJ homolog subfamily C member 11 | 5 | 0.17 | 0.12 | 0.012 | 0.75 |
| O75746 | CMC1 | Calcium-binding mitochondrial carrier protein Aralar1 | 9 | 0.13 | 0.15 | 0.012 | 1.13 |
| Q9UJ68 | MSRA | Mitochondrial peptide methionine sulfoxide reductase | 2 | 0.93 | 0.85 | 0.012 | 0.92 |
| Q9H9B4 | SFXN1 | Sideroflexin-1 | 9 | 0.14 | 0.11 | 0.012 | 0.82 |
| P05165 | PCCA | Propionyl-CoA carboxylase alpha chain, mitochondrial | 12 | 0.35 | 0.42 | 0.013 | 1.21 |
| O95169 | NDUB8 | NADH dehydrogenase [ubiquinone] 1 beta subcomplex subunit 8, mitochondrial | 5 | 0.18 | 0.13 | 0.014 | 0.72 |
| P49411 | EFTU | Elongation factor Tu, mitochondrial | 22 | 0.22 | 0.26 | 0.014 | 1.20 |
| P53597 | SUCA | Succinyl-CoA ligase [ADP/GDP-forming] subunit alpha, mitochondrial | 7 | 0.69 | 0.92 | 0.015 | 1.34 |
| O95168 | NDUB4 | NADH dehydrogenase [ubiquinone] 1 beta subcomplex subunit 4 | 4 | 0.32 | 0.26 | 0.016 | 0.80 |
| Q9Y305 | ACOT9 | Acyl-coenzyme A thioesterase 9, mitochondrial | 8 | 0.62 | 0.75 | 0.017 | 1.20 |
| P18859 | ATP5J | ATP synthase-coupling factor 6, mitochondrial | 6 | 0.13 | 0.11 | 0.017 | 0.89 |
| P56385 | ATP5I | ATP synthase subunit e, mitochondrial | 4 | 0.19 | 0.17 | 0.017 | 0.88 |
| Q9Y5J7 | TIM9 | Mitochondrial import inner membrane translocase subunit Tim9 | 2 | 0.22 | 0.17 | 0.017 | 0.78 |
| P47985 | UCRI | Cytochrome b-c1 complex subunit Rieske, mitochondrial | 9 | 0.10 | 0.08 | 0.017 | 0.81 |
| P21796 | VDAC1 | Voltage-dependent anion-selective channel protein 1 | 16 | 0.10 | 0.09 | 0.018 | 0.86 |
| Q9ULC5 | ACSL5 | Long-chain-fatty-acid--CoA ligase 5 | 4 | 0.02 | 0.03 | 0.018 | 1.41 |
| Q5XKP0 | QIL1 | Protein QIL1 | 3 | 0.20 | 0.14 | 0.018 | 0.72 |
| O95140 | MFN2 | Mitofusin-2 | 13 | 0.18 | 0.16 | 0.018 | 0.89 |
| P00390 | GSHR | Glutathione reductase, mitochondrial | 5 | 0.09 | 0.12 | 0.018 | 1.32 |
| Q07812 | BAX | Apoptosis regulator BAX | 3 | 0.08 | 0.09 | 0.018 | 1.09 |
| P45880 | VDAC2 | Voltage-dependent anion-selective channel protein 2 | 15 | 0.11 | 0.09 | 0.021 | 0.81 |
| P52815 | RM12 | 39S ribosomal protein L12, mitochondrial | 4 | 0.20 | 0.18 | 0.021 | 0.92 |
| P43155 | CACP | Carnitine O-acetyltransferase | 8 | 4.31 | 6.40 | 0.022 | 1.48 |
| P51970 | NDUA8 | NADH dehydrogenase [ubiquinone] 1 alpha subcomplex subunit 8 | 5 | 0.11 | 0.08 | 0.022 | 0.74 |
| O75251 | NDUS7 | NADH dehydrogenase [ubiquinone] iron-sulfur protein 7, mitochondrial | 3 | 0.13 | 0.09 | 0.023 | 0.69 |
| Q9BPW8 | NIPS1 | Protein NipSnap homolog 1 | 3 | 0.27 | 0.19 | 0.023 | 0.70 |
| Q9H845 | ACAD9 | Acyl-CoA dehydrogenase family member 9, mitochondrial | 5 | 0.47 | 0.39 | 0.024 | 0.83 |
| O96008 | TOM40 | Mitochondrial import receptor subunit TOM40 homolog | 5 | 0.13 | 0.15 | 0.027 | 1.15 |
| P56134 | ATPK | ATP synthase subunit f, mitochondrial | 2 | 0.07 | 0.05 | 0.027 | 0.78 |
| P54819 | KAD2 | Adenylate kinase 2, mitochondrial | 14 | 3.54 | 3.74 | 0.028 | 1.06 |
| Q9NS69 | TOM22 | Mitochondrial import receptor subunit TOM22 homolog | 3 | 0.04 | 0.04 | 0.028 | 1.19 |
| P11182 | ODB2 | Lipoamide acyltransferase component of branched-chain alpha-keto acid dehydrogenase complex, mitochondrial | 6 | 0.14 | 0.27 | 0.029 | 1.87 |
| O43920 | NDUS5 | NADH dehydrogenase [ubiquinone] iron-sulfur protein 5 | 4 | 0.13 | 0.09 | 0.030 | 0.70 |
| O43678 | NDUA2 | NADH dehydrogenase [ubiquinone] 1 alpha subcomplex subunit 2 | 3 | 0.16 | 0.18 | 0.032 | 1.09 |
| Q8WY22 | BRI3B | BRI3-binding protein | 2 | 0.03 | 0.04 | 0.032 | 1.35 |
| Q9NZ45 | CISD1 | CDGSH iron-sulfur domain-containing protein 1 | 2 | 0.11 | 0.09 | 0.033 | 0.80 |
| P31040 | SDHA | Succinate dehydrogenase [ubiquinone] flavoprotein subunit, mitochondrial | 15 | 0.28 | 0.31 | 0.033 | 1.11 |
| P83111 | LACTB | Serine beta-lactamase-like protein LACTB, mitochondrial | 3 | 0.22 | 0.25 | 0.034 | 1.13 |
| O95202 | LETM1 | LETM1 and EF-hand domain-containing protein 1, mitochondrial | 13 | 0.14 | 0.12 | 0.036 | 0.86 |
| P35232 | PHB | Prohibitin | 10 | 0.11 | 0.08 | 0.037 | 0.73 |
| Q9P0J1 | PDP1 | [Pyruvate dehydrogenase [acetyl-transferring]]-phosphatase 1, mitochondrial | 6 | 0.41 | 0.53 | 0.037 | 1.32 |
| Q16740 | CLPP | Putative ATP-dependent Clp protease proteolytic subunit, mitochondrial | 4 | 0.25 | 0.40 | 0.039 | 1.61 |
| Q02338 | BDH | D-beta-hydroxybutyrate dehydrogenase, mitochondrial | 5 | 0.15 | 0.12 | 0.040 | 0.83 |
| Q8IXI1 | MIRO2 | Mitochondrial Rho GTPase 2 | 4 | 0.17 | 0.15 | 0.040 | 0.86 |
| P30044 | PRDX5 | Peroxiredoxin-5, mitochondrial | 10 | 0.46 | 0.42 | 0.042 | 0.92 |
| Q9BPX6 | MICU1 | Calcium uptake protein 1, mitochondrial | 9 | 0.28 | 0.31 | 0.046 | 1.11 |

Abbreviations: a) Peptides used for quantitation; b) p-value; c) fold CFS/healthy.
