# Supplementary material for: Bottom-up proteomics suggests an association between differential expression of mitochondrial proteins and chronic fatigue syndrome
Source: Transl Psychiatry. 2016 Sep 27;6(9):e904–. doi: 10.1038/tp.2016.184 (PMC5048217; doi:10.1038/tp.2016.184)
Supplement: Supplementary Table S2 [file tp2016184x5.doc]

|  | **Table S2. List of the most significant “biological functions” associated with our proteins obtained with Signaling pathway analysis.** | | |  |
| --- | --- | --- | --- | --- |
|  | **Diseases or Functions Annotation** | **p-value** | **Gene name of molecules** |  |
|  | Metabolism of Isocitric Acid | 1.83e-08 | ACO2,IDH3A,IDH3B |  |
|  | Metabolism of NADH | 4.00e-07 | IDH3A,IDH3B,MDH2 |  |
|  | Metabolism of Nucleic Acid Component  or Derivative | 8.15e-07 | ACAA2,ACADSB,ALDH6A1,ATP5B,HMGCL,  IDH3A,IDH3B,MDH2 |  |
|  | Catabolism of Valine | 1.56e-06 | ALDH6A1,HIBADH |  |
|  | Beta-Oxidation of Fatty Acid | 2.60e-06 | ACAA2,ACADM,ACADSB,ECHS1 |  |
|  | Oxidation of Fatty Acid | 3.55e-06 | ACAA2,ACADM,ACADSB,ECHS1,GOT2 |  |
|  | Fatty Acid oxidation Disorder | 4.67e-06 | ACADM,ETFA,OXCT1 |  |
|  | Metabolism of Purine Nucleotide | 7.69e-06 | ATP5B,IDH3A,IDH3B,MDH2 |  |
