# Supplementary material for: Bottom-up proteomics suggests an association between differential expression of mitochondrial proteins and chronic fatigue syndrome
Source: Transl Psychiatry. 2016 Sep 27;6(9):e904–. doi: 10.1038/tp.2016.184 (PMC5048217; doi:10.1038/tp.2016.184)
Supplement: Supplementary Table S3 [file tp2016184x6.doc]

|  | **Table S3.** **List of the 3 most significant “biological functions” with identified proteins ranked by p-value.** | | | | |  |
| --- | --- | --- | --- | --- | --- | --- |
|  | **GENE** | **ID** | **Top ranked biological functions** | **p-value** | **fold** |  |
|  | *Metabolism of Isocitric Acid* | | | | |  |
|  | IDH3B | O43837 | Isocitrate dehydrogenase [NAD] subunit beta, mitochondrial | 0.00051 | 2.4 |  |
|  | ACO2 | Q99798 | Aconitate hydratase. mitochondrial | 0.00140 | 2.2 |  |
|  | IDH3A | P50213 | Isocitrate dehydrogenase [NAD] subunit alpha. mitochondrial | 0.00308 | 2.3 |  |
|  | *Metabolism of NADH* | | | | |  |
|  | IDH3B | O43837 | Isocitrate dehydrogenase [NAD] subunit beta. mitochondrial | 0.00051 | 2.4 |  |
|  | MDH2 | P40926 | Malate dehydrogenase. mitochondrial | 0.00125 | 2.3 |  |
|  | IDH3A | P50213 | Isocitrate dehydrogenase [NAD] subunit alpha. mitochondrial | 0.00308 | 2.3 |  |
|  | *Metabolism of Nucleic Acid Component* | | | | |  |
|  | ACAA2 | P42765 | 3-ketoacyl-CoA thiolase. mitochondrial | 0.00018 | 4.2 |  |
|  | ATP5B | P06576 | ATP synthase subunit beta. mitochondrial | 0.00044 | 2.1 |  |
|  | IDH3B | O43837 | Isocitrate dehydrogenase [NAD] subunit beta. mitochondrial | 0.00051 | 2.4 |  |
|  | MDH2 | P40926 | Malate dehydrogenase. mitochondrial | 0.00125 | 2.3 |  |
|  | HMGCL | P35914 | Hydroxymethylglutaryl-CoA lyase. mitochondrial | 0.00145 | 2.3 |  |
|  | ACADSB | P45954 | Short/branched chain specific acyl-CoA dehydrogenase. mitochondrial | 0.00163 | 2.2 |  |
|  | IDH3A | P50213 | Isocitrate dehydrogenase [NAD] subunit alpha. mitochondrial | 0.00308 | 2.3 |  |
|  | ALDH6A1 | Q02252 | Methylmalonate-semialdehyde dehydrogenase [acylating]. mitochondrial | 0.00398 | 2.1 |  |
